# Phylogeographic Reconstruction of African Yellow Fever Virus Isolates Indicates Recent Simultaneous Dispersal into East and West Africa

**DOI:** 10.1371/journal.pntd.0001910

**Published:** 2013-03-14

**Authors:** Andrew Beck, Hilda Guzman, Li Li, Brett Ellis, Robert B. Tesh, Alan D. T. Barrett

**Affiliations:** 1 Department of Pathology, Center for Biodefense and Emerging Infectious Diseases, Sealy Center for Vaccine Development, Institute for Human Infections and Immunity, and Center for Tropical Diseases, University of Texas Medical Branch, Galveston, Texas, United States of America; 2 Duke-NUS Graduate Medical School, Singapore; Duke-NUS, Singapore

## Abstract

Yellow fever virus (YFV) is a mosquito-borne flavivirus that is a major public health problem in tropical areas of Africa and South America. There have been detailed studies on YFV ecology in West Africa and South America, but current understanding of YFV circulation on the African continent is incomplete. This inadequacy is especially notable for East and Central Africa, for which the unpredictability of human outbreaks is compounded by limitations in both historical and present surveillance efforts. Sparse availability of nucleotide sequence data makes it difficult to investigate the dispersal of YFV in these regions of the continent. To remedy this, we constructed Bayesian phylogenetic and geographic analyses utilizing 49 partial genomic sequences to infer the structure of YFV divergence across the known range of the virus on the African continent. Relaxed clock analysis demonstrated evidence for simultaneous divergence of YFV into east and west lineages, a finding that differs from previous hypotheses of YFV dispersal from reservoirs located on edges of the endemic range. Using discrete and continuous geographic diffusion models, we provide detailed structure of YFV lineage diversity. Significant transition links between extant East and West African lineages are presented, implying connection between areas of known sylvatic cycling. The results of demographic modeling reinforce the existence of a stably maintained population of YFV with spillover events into human populations occurring periodically. Geographically distinct foci of circulation are reconstructed, which have significant implications for studies of YFV ecology and emergence of human disease. We propose further incorporation of Bayesian phylogeography into formal GIS analyses to augment studies of arboviral disease.

## Author Summary

Yellow fever virus (YFV) is a mosquito-transmitted pathogen of great public health significance, which is endemic to tropical areas of Africa and South America. Despite the availability of an effective vaccine, and programs that exist in many endemic areas to reduce populations of mosquitoes, YFV continues to circulate and emerge in regions with developing public health infrastructures. Periodic outbreaks of YFV into humans are unpredictable and merit thorough investigation of the ecology and genetic diversity of the virus. Our analyses improve the current understanding of African YFV evolution in several respects. We have included unpublished viral sequence data from Central and East Africa, which is significant because the availability of YFV isolates from these regions is extremely limited. We present a modeled geographic structure of African YFV dispersal, and propose a new model for the spread of YFV based on concurrent historical movement of the virus from reservoirs in central African jungles to both eastern and western regions of the continent. Our results provide evidence for the presence of unique genotypes of the virus in both central and east African circulation. The presented findings not only provide insight to estimations of outbreak risk for the regions in question, but also contribute to rational GIS analysis and approaches to vaccination campaigns.

## Introduction

Yellow fever virus (YFV) is a mosquito-vectored member of the family *Flaviviridae*, genus *Flavivirus*, which includes the causative agents of dengue fever, Japanese encephalitis, West Nile fever, and other prominent, arthropod-borne infections. The enveloped, 50 nm particle, encloses a single-stranded, positive-sense RNA genome of approximately 10.8 kb, bearing a 5′ cap, and 5′ and 3′ terminal untranslated regions (UTRs), without 3′ polyadenylation. Translation occurs as a single open reading frame, the product of which is co- and post-translationally cleaved into 10 functional proteins. The three structural proteins, capsid (C), pre-membrane/membrane (prM/M), and envelope (E), are upstream of 7 nonstructural proteins NS1, NS2A, NS2A, NS3, NS4A, NS4B, and NS5. Many members of the genus *Flavivirus* participate in complex transmission cycles that involve mammalian and insect hosts [1]. The risk of periodic emergence into humans depends upon many factors, including vaccination status, elevation, dispersal patterns, and interaction with competent vector populations.

Geographic distribution of YFV endemicity covers tropical areas of Africa and South America. For the African continent, human epidemics have been reported from western regions with more regular frequency than the east. This observation is almost certainly biased by regional differences in population density, vaccination coverage, and surveillance capacity [2]. However, ecological factors presumably govern some observed differences in outbreak severity and frequency of East and West African YFV outbreaks. The natural history of human YFV infection includes periodic emergence events in rainforest perimeter transition zones, following contact of humans with infected hematophagous mosquitoes of the *Aedes* genus. Prediction of YFV emergence in East and Central Africa is confounded both by overlapping distributions of competent vectors and uncertain dynamics of sylvatic maintenance in populations of arboreal nonhuman primates. Sylvatic vector burden for the region in question is dominated by the diverse *A. africanus* complex, which ranges across the entirety of sub-Saharan Africa. Regardless of the structure of maintenance cycles, proximity to contiguous vector habitat is a prime risk factor of infection for susceptible human populations, an ecological property originally supported by high rates of seroprevalence in residents of Ugandan villages adjoining forest galleries of known YFV endemicity [3]. Viral dispersal patterns may have tracked with human movement as a consequence of civil unrest, or with agricultural and population changes brought by the European colonial era [4]. The displacement of north Ugandan citizens into temporary camps during civil conflicts of the previous two decades is a potential example of such an alteration to the YFV transmission landscape [5].

Nucleotide sequence analysis of 3′UTRs of representative YFV genomes have inferred a shared ancestry for West and East/Central African genotypes of the virus, based on the variable presence of one, two or three 41-nucleotide repeat segments, which presumably form secondary RNA structures that contribute to host range adaptation [6], [7]. Overall, phylogenetic and epidemiological analysis of the *Flaviviridae* supports a model of YFV emergence that is dependent upon both the presence of competent vectors and sylvatic nonhuman primate reservoir hosts [8], [9].

Sampling history for YFV in Africa is limited, especially with respect to central and eastern regions of the continent. However, phylogenies derived from the use of the historical YFV isolates have yielded important data on the association of viral dispersal patterns with ecological features, including evidence for the existence of geographically associated YFV genotypes. The first phylogenetic evidence for geographic association of African YFV taxa resolved the bifurcation of African YFV circulation into east and west lineages [10]. Variable presence of repeat segments in the 3′ noncoding regions of YFV isolates was associated with the region of isolation (East/West Africa and South America), indicating a role for repeat element structures in virus range adaptation [6], [11]. A neighbor-joining analysis of African YFV strains, provided evidence for the presence of 5 prototype lineages in circulation in the continent, West Africa I, West Africa II, East Africa, East/Central Africa, and Angola [12]. Subsequent analysis using full-length YFV sequences provided confirmatory evidence for the putative geographic structure [13].

Bayesian estimation of divergence dates offers clarity to historical correlations of virus dispersal. Utilization of accurate clock models for arboviral phylogeny estimation is a matter of ongoing study. The reliability of this technique for measurements of viral evolutionary dynamics depends upon a combination of historical reference events, confirmation by independent data, and uncertainty of posterior estimates. In this manner, Bryant and colleagues estimated a plausible timescale of YFV divergence events, finding experimental support for the hypothesized introduction of YFV from West Africa to South America with the transatlantic slave trade [14]. Subsequently, relaxed clock analysis was used to estimate divergence histories for Trinidadian YFV isolates, producing evidence for enzootic maintenance of YFV on the island [15]. Significance of estimated dates was derived by correlation with observed recent history of YFV epizootic activity in nonhuman primate populations. A relaxed clock analysis was performed to determine phylogenetic placement of new sequence data from a Ugandan YFV outbreak that had occurred in 2010 [16]. This rare acquisition of east African sequence data resulted in a tree of topology and time estimates consistent with other published materials, using a combination of envelope gene and full-length sequences.

Phylogeographic modeling incorporates distance and location under the presumption that these features are informative properties of the viral dispersal path. Most significantly, these methods permit direct testing of hypotheses on lineage dispersal and geographic association, including estimation of surface diffusion rates [17]. We believe that use of diffusion analysis is justified for reconstruction of African YFV phylogenies due to both the origin of sequence isolations from a contiguous landmass, and epidemiological guidance that continuous vegetation is a useful proxy for YFV infection risk [18]. The spatial relationships between extant African YFV lineages are currently unknown [4]. Determination of lineage orientation in physical space offers clarity to the structure of periodic and uncertain disease emergence, especially for isolates of east African origin. Our dataset includes a recent historical phylogeny of African YFV ranging in time of isolation from the prototype strain Asibi, isolated in 1927, to a recent isolate from 2010 [16]. These analyses present the most complete Bayesian phylogeny of African YFV to date.

## Methods

### Sequences

49 partial coding region YFV sequences comprising 670 nucleotides spanning prM, M and E genes were used in the study. Publicly available sequences were downloaded from NCBI/Genbank (Table S1a). Seven novel Central and East African YFV isolates were obtained from the World Reference Center for Emerging Viruses and Arboviruses (Galveston, TX) amplified by RT-PCR, sequenced for the specified region, and added to the alignment. Sequences newly added to GenBank are as follows: JX012097, JX012098, JX012099, JX012100, JX012101, JX012102, JX012103. The complete listing of accession taxa used in the study are compiled as supplemental data (Table S1).

Amplification conditions were as follows: Reverse transcription was performed at 50°C for 30 min, followed by denaturation at 95°C for 2 min.; 40 amplification cycles were performed at 95°C for 10 s, 55°C for 30 s, 68°C for 60 s. Final extension was at 68°C for 2 min. Reactions were cooled to 4°C before downstream use. Primers used for all amplifications were CAG (GGTGTCCCGACTCAATGGAA) and YF7 (CCAAAGAGCCCACAACCATT) as described previously [12]. Amplified fragments were agarose gel-purified and directly sequenced. All sequencing was performed at the Protein Chemistry Core Laboratory at the University of Texas Medical Branch (Galveston, TX). Alignment was performed following translation using the MUSCLE algorithm as implemented in the SEAVIEW v.4 platform [19], [20]. The prepared alignment was screened for recombination with methods RDP, GENECONV, BOOTSCAN, and 3SEQ using the RDP3 platform [21]–[25]. Using a consensus of these methods, we found no significant evidence of recombination in the alignment.

### Phylogenetic Inference

Bayesian Markov-Chain Monte Carlo (MCMC) analyses were performed using the BEAST 1.6.1 platform on the CIPRES computational resource (CA, University of California San Diego) [26], [27]. A codon-based substitution model SRD06 was used for all inference calculations in the study [28]. A chain length of 30 million generations was used, discarding burn-in of 10 percent. Stable combinations of molecular clock and coalescent model were modeled, and assessed for relative significance by comparisons of marginal likelihood estimated by path-sampling and stepping stone sampling [29]. A consensus of these techniques supported the use of a lognormally distributed clock with a constant population coalescent prior. A Bayesian skyline coalescent prior was applied to a lognormally distributed clock model to obtain estimates of population change for the dataset. Analysis of posterior data, including assessment of convergence and Bayes factor (BF) comparisons, was performed using Tracer v1.5 [30]. Posterior results were summarized as maximum clade credibility (MCC) trees with TreeAnnotator v. 1.6.1, and visualized using FigTree v.1.3.1. Confirmatory inference was performed using 20 partial coding sequences containing the C-terminal section of the NS5 gene and 3′ non-coding region (786 nucleotides). 3′ noncoding region sequences were aligned using the ClustalW algorithm and hand-verified using the BioEdit platform [31]. A neighbor-joining tree using observed distance and 1000 bootstrap replicates was computed for this alignment using SEAVIEW v.4.

### Phylogeographic Inference

The MCMC chain was sampled for discrete state, bivariate continuous, and relaxed random walk (RRW) diffusion as implemented in BEAST v.1.6.1. Bayesian stochastic search variable selection (BSSVS) was used to provide evidence for statistically supported diffusion between state variables. BSSVS output and surfaces representing uncertainty for continuous diffusion processes were formatted as KML using the SPREAD utility [32]. Coordinate determination for taxon locations was performed using Google Earth v.6.0.1. For final rendering, KML files were imported and manipulated in ArcMap 10.0 [ESRI, Redlands CA] using an Albers equal-area conic projection for the African continent. Surfaces representing uncertainty for the diffusion process were observed as overlay with a maximum entropy raster considering the presence of *Ae. africanus*; the raster file was obtained from the MosquitoMap resource of the Walter Reed Biosystematics Unit [Suitland, MD] [33].

#### Preparation of Discrete State Analyses

Taxon states were sampled according to the country of isolation (K = 14). BSSVS sampling was additionally performed using distance-informed priors to apply relative penalty to state transitions representing greater geographic distance. Bayes factors greater than 5 and BSSVS indicators greater than 0.5 were used as criteria for state transition significance. Final projection and visualization were accomplished using country boundary centroid coordinates.

#### Preparation of Diffusion Analyses

For West African isolates, we retrieved location data from the outbreak histories of Digoutte [34]. For East African isolates, we utilized location data in decimal degrees of latitude/longitude for all sampling locations as listed in the historical YFV outbreak summary of Ellis and Barrett [4]. Political boundary centroid coordinates were used for isolates without available detail. Diffusion analysis was modeled using continuous, Cauchy, lognormal, and gamma-distributed diffusion rates, and posterior results were assessed for relative significance using Bayes Factor comparisons of marginal likelihood.

### Selection Analyses

We performed selection analyses on the full dataset using Datamonkey server implementation of HyPhy [35]. We used the Single Likelihood Ancestor Counting (SLAC), Fixed Effects Likelihood (FEL), Internal Fixed-Effects Likelihood (IFEL), and Random-Effects Likelihood (REL) algorithms to assay for the presence of selected sites in the prM/E alignment. Significance of SLAC, FEL and IFEL results used a p-value cutoff of 0.05, and results of REL were assessed to be significant for Bayes Factors greater than 50.0.

## Results

### Addition of Nucleotide Sequence Data for East Africa

Nucleotide sequence information for African YFV is sparse, especially for eastern and central regions of the continent. We acquired and sequenced 7 unpublished YFV sequences fragments, now totaling 26 isolates from Central and East Africa, and comprising all known isolates from this region. Although the complete dataset remains sparse, requiring careful interpretation, the resultant phylogeny provides new information on regional dispersal patterns of YFV, supported by several methods of Bayesian phylogeographic inference.

### Tree Topology

**Figure 1:**
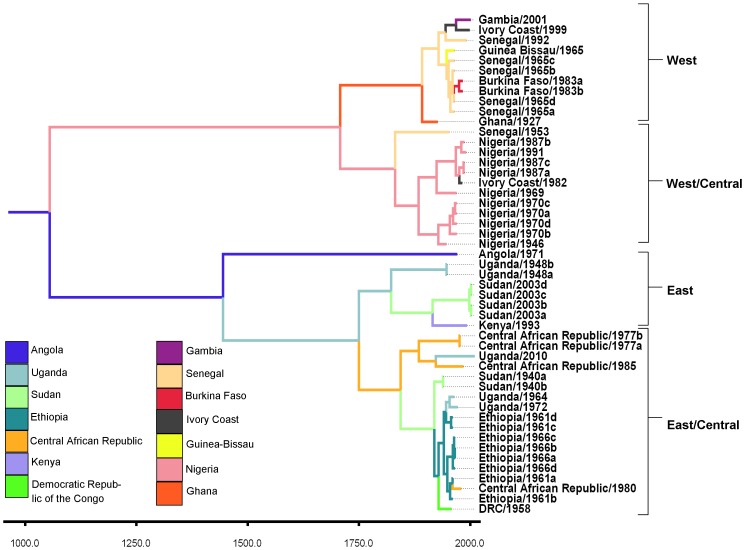
Phylogenetic tree containing results of the discrete state model. Maximum clade credibility phylogeny for YFV under the discrete state transition model. Legend color indicates highest probability state transition for annotated node.

The maximum clade credibility tree (Figure 1) supports the presence of two primary circulating lineages in western and eastern regions of the African continent. Each lineage is further bifurcated spatially into outlying and central genotypes (Figure 1). Posterior support was high for ancestral nodes of the primary lineages and subordinate genotypes (Table 1). Confirmatory neighbor-joining analysis based on the NS5-3′UTR sequences (Figure S1; Taxa listed in Table S1b) was found to have the same geographic topology as the Bayesian phylogeny constructed from the prM/E sequences (Figure 1).

**Table 1:** Divergence estimates for major lineages.

Node	Year	Low HPD	High HPD	Posterior
MRCA	1007	342	1608	1.0
West Lineage	1695	1520	1853	1.0
East Lineage	1736	1579	1872	1.0
East Lineage and Angola	1415	1017	1769	1.0
West Genotype	1887	1840	1922	1.0
West/Central Genotype	1820	1728	1902	1.0
East/Central Genotype	1813	1694	1917	0.9
East Genotype	1835	1740	1912	1.0

The table contains estimates of divergence times, in decimal calendar years CE, for mean heights of significant nodes in the Bayesian phylogeny. HPD intervals and posterior node probability are included. These results were obtained using a constant population demographic prior and relaxed, lognormally-distributed clock.

### Divergence Times

Divergence dates inferred by models under Bayes factor support were tabulated with accompanying 95% highest posterior density (HPD) intervals (Table 1). The branch time-corrected mutation rate was 2.8×10^−4^ substitutions/site/year, 95%HPD: 1.3×10^−4^ to 4.5×10^−4^ substitutions/site/year. In order from earliest inferred divergence and expressed as mean height in decimal years for the indicated node, the time of the most recent common ancestor (TMRCA) was dated to 1007 CE (95% HPD: 342 to 1608). The divergence of the singular Angola isolate from the East lineage was dated to 1415 CE (95% HPD: 1017 to 1769). The West lineage was dated to 1695 CE (95% HPD: 1520 to 1853) with the west/central genotype dated to 1820 CE (95% HPD: 1728 to 1902) and the west genotype dated to 1887 CE (95% HPD: 1840 to 1922). The East lineage was dated to 1844.37 CE (95% HPD: 1751 to 1909) with the east/central genotype dated to 1736 CE (95% HPD: 1579 to 1872) and the east genotype dated to 1835 CE (95% HPD: 1740 to 1912).

### Selection and Demographic Analyses

Analysis using the SLAC algorithm detected a global ratio of nonsynonymous to synonymous nucleotide substitutions (*d_N_/d_S_*) of 0.0382, which suggests the presence of predominantly purifying selection. Integrated results from all algorithms used found evidence for 192 sites under negative selection. FEL analysis detected evidence for one positively selected site, position 631 of the alignment, an envelope protein amino acid substitution G100S that was present in three isolates: Democratic Republic of Congo/1958, Guinea-Bissau/1965, and Senegal/1992 (p = 0.045). REL analysis also detected positive selection at this site (BF = 55.43). SLAC and IFEL analyses recovered no evidence of positively selected sites.

**Figure 2:**
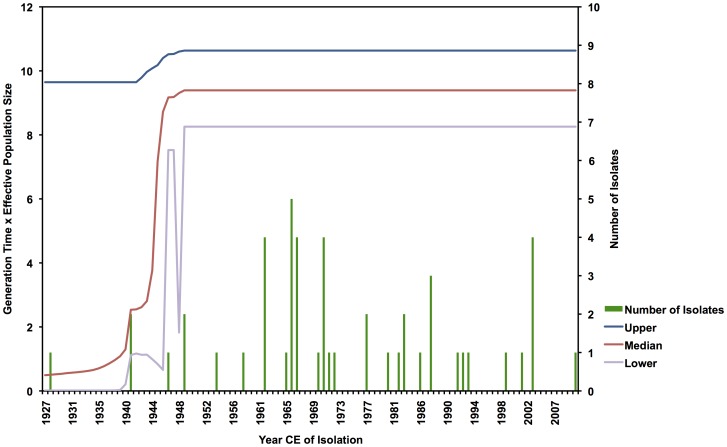
Bayesian skyline demographic analysis for the prM/E alignment. Plot is a trace of the effective population size over time of the YFV alignment, using a Bayesian skyline model. Mean population size (black line) is displayed with upper and lower bounds of the 95% HPD interval (blue and purple lines). Constant value of the trace between years 1940 and 2010 is indicative of stability in the estimated viral population during that time.

Bayesian skyline analysis indicated a stable viral population over the length of time represented by the sample set (Figure 2). The early terminus of the plot, encompassing the date ranges of 1927–1940, lacks adequate sampling frequency to resolve the skyline model at these timepoints, but the time period of the sample between 1940 and 2010 is adequately sampled and shows evidence of an unchanging population size during that period. Lack of sampling in some intervals (see Figure 2) and the sensitivity of this coalescent model to short sequence length requires that any interpretation should be made with caution.

### Phylogeographic Analyses

**Figure 3:**
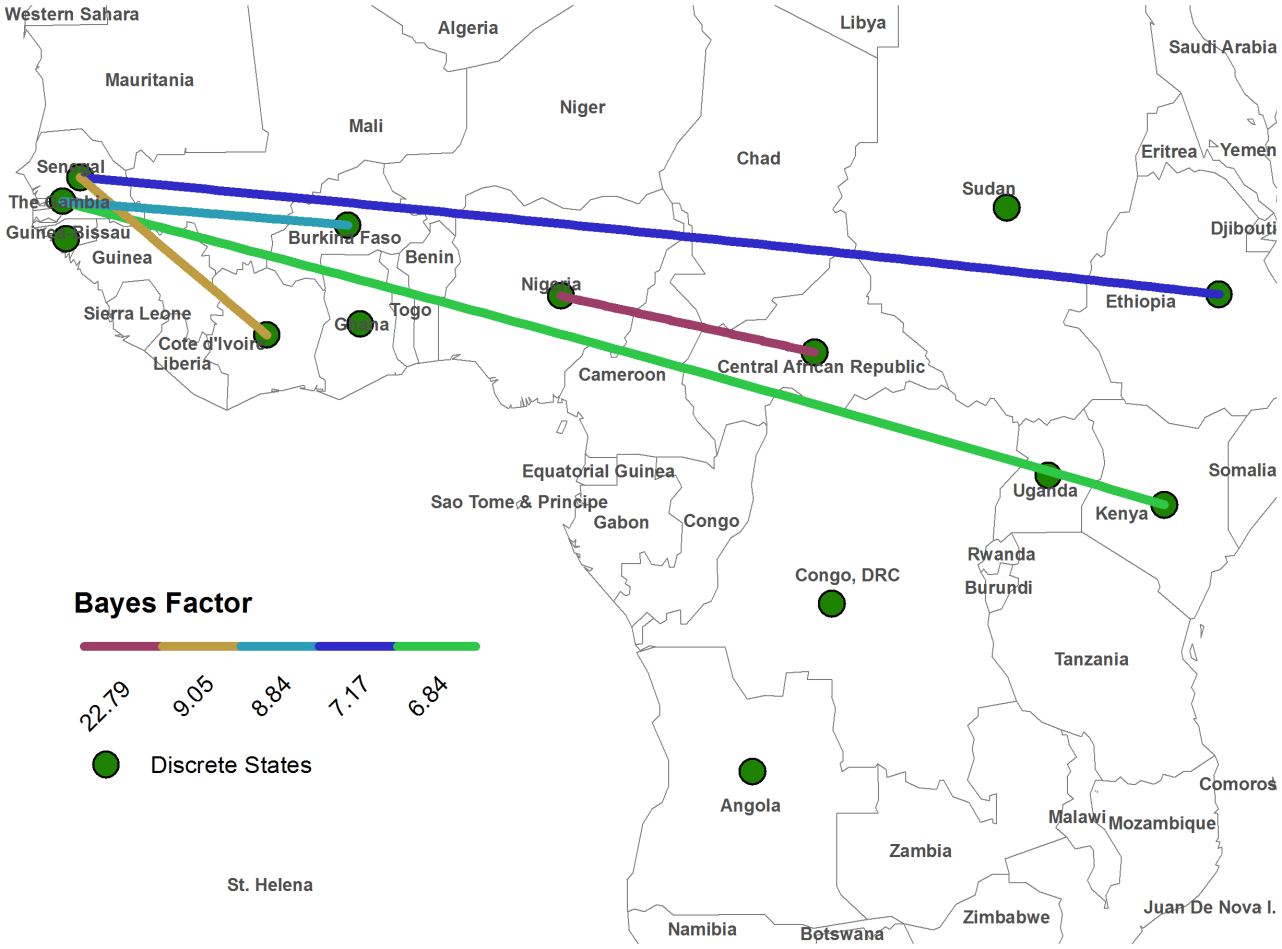
Map of supported state transitions for Bayesian Stochastic Search Variable Test (BSSVS). Colored lines represent the transitions that were statistically supported in the inferred phylogeny, using an uncorrected model. Significance was assessed for state transitions resulting in a Bayes factor greater than 5 and BSSVS indicator value greater than .50.

Discrete state analysis returned a number of dominant transitions in the posterior sample, indicating focal dominance of Ethiopian, Ugandan, Nigerian, and Senegalese origin in four well supported and geographically associated clades, respectively (Figure 1). The highest posterior probability for the root state was Angola (0.083). BSSVS models returned significant BF values for a number of state transitions. Under the criteria (BF>5, Indicator>0.5), significance for certain traits was maintained with the use of a linearly relative distance penalty (Table 2). All significant transitions returned from the BSSVS model span the longitudinal axis of the African continent, connecting the east and west lineages (Figure 3). The transition between Nigeria and the Central African Republic received the greatest relative support by the BF criterion (BF = 22.79, uncorrected).

**Table 2:** Bayes factors for state transitions, comparing significance output for uncorrected vs. distance-penalized connections.

	Bayes Factors			
Transition Pair	Uncorrected	Indicator	Distance Penalized	Indicator
Central African Republic – Nigeria	22.79	0.80	17.91	0.76
Senegal – Ivory Coast	9.05	0.62	10.17	0.64
Gambia – Burkina Faso	8.84	0.61	10.38	0.64
Ethiopia – Senegal	7.17	0.56	7.15	0.56
Kenya – Gambia	6.85	0.55	5.42	0.49

* **Transition pair significance for indicator value criteria was not maintained between the uncorrected and distance-penalized model.**

Significant reversible state transitions for BSSVS test for Bayes factors greater than 3 and indicator value greater than .50. Table shows a comparison between factor values obtained with an uncorrected model, and a set obtained by imposing an increasing linear distance penalty on computed distance between states. Under distance penalty, significance was reduced below indicator value criterion for indicated transition pairs.

**Figure 4:**
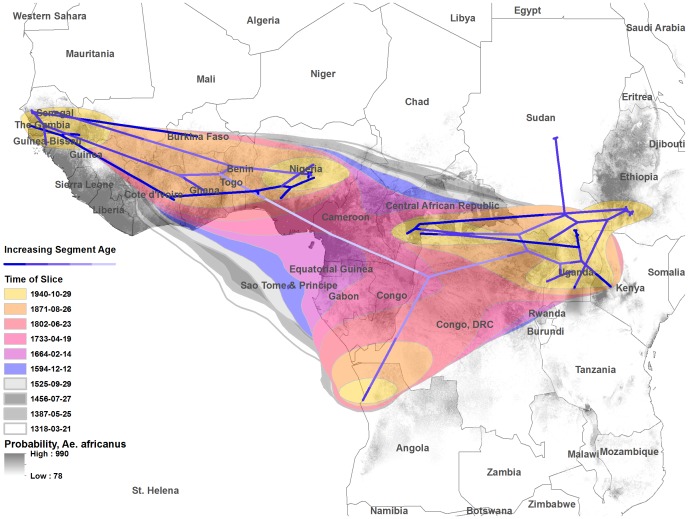
Map of the African continent containing projection of the tree and uncertainty for Cauchy-distributed random walk diffusion. The inferred phylogeny is projected along a time gradient, with mean root location and results of the diffusion analysis. Colored polygon surfaces represent 80% HPD uncertainty for the location of estimated nodes, using Cauchy-distributed diffusion rates for ten equal time intervals along the length of the tree. Polygons are overlaid with a maximum entropy raster considering the presence of *Aedes africanus* on the African continent.

Cauchy-distributed diffusion rates were highly supported by BF comparisons of all available models (Table S2). For Cauchy-distributed RRW diffusion, the mean root position was inferred at 4.2°N, 10.5°E (Central Cameroon). Mean lateral diffusion rate was 10.6 km/yr, 95% HPD = 4.9 to 17.4 km/yr. The 80% HPD interval containing diffusion uncertainty coverage for the most recent common ancestor of the tree encompasses a broad region of the mass of the African continent (Figure 4, grey polygons).

## Discussion

Two West African genotypes were resolved as described previously (West Africa I and West Africa II) [12]. Based on the current findings we redefine West Africa I and West Africa II genotypes as the West/Central genotype and Western genotype, respectively. The West/Central genotype is defined by predominant sampling of isolates of Nigerian origin while the Western genotype is defined by isolates originating primarily from Senegal, Guinea-Bissau, and the Gambia. Incorporation of sequence information from seven additional isolates in this study enabled us to find evidence for structure in the East African lineage that has so far been poorly resolved. As presented, the inferred phylogeny supports the presence of both East and East/Central genotypes with high posterior probability. The proposed East/Central genotype is defined primarily by sampling from Ethiopia and the Democratic Republic of the Congo. The proposed east genotype is represented by isolates primarily recovered from Sudan and Uganda. Results from multiple independent models were used to support claims of geographic structure in the dataset.

Posterior estimates for time structure of the dataset are consistent with patterns observed in previous studies. The mean substitution rate estimate of 2.8×10^−4^ substitutions/site/year, (95%HPD 1.3 to 4.5×10^−4^ substitutions/site/year) overlaps a previously estimated range 1.0×10^−4^ to 3.3×10^−4^ substitutions/site/year, (mean = 2.1×10^−4^) computed from an alignment of complete E gene sequences [36]. The estimated rate from this study is not directly comparable to that of Sall et al. [36], as the alignments used are derived from different regions of the YFV genome. However, the overlap of posterior density does not permit significant discrimination of the means derived from either study. Additionally, our estimation is similar to that provided by Auguste, et al., using the criterion of 95% HPD overlap and based primarily on isolates from the Americas [15].

Comparison of dates for significant nodes shows broad agreement with previous findings. The common ancestor of all African lineages was estimated to have arisen in 1003 years before the most recent taxon, a later divergence than proposed by Bryant et al. (742) [14], but earlier than that proposed by Sall et al. (1262) [36], or McMullan et al. (2188) [37], however, overlap of posterior density is considerable for estimations of Bryant et al [38] and Sall et al. [36], indicating agreement.

Estimations of divergence dates were indistinguishable for the common ancestors of both east and west lineages, potentially indicating simultaneous dispersal of these lineages to the eastern and western regions of the African continent from ancestral populations. Most significantly, ancestral nodes of the subordinate genotypes were estimated to have arisen in a spatially biased sequence, with earlier nodes belonging to the east/central and west/central genotypes. This finding suggests that spatially outlying genotypes were the most recently established, and that ancestral populations of YFV were located centrally on the continent. Sall et. al. [36] inferred a topology in which YFV taxa isolated from the Central African Republic are represented in both east and west lineages, implying a pattern of multiple YFV introductions from central reservoirs. Across the phylogeny, several instances of relatively high substitution rates are recovered in the earliest-emerged taxa of several clades (Nigeria/1948, Uganda/1948, Central African Republic/1977, Kenya/1993) (Figure S2). The appearance of these higher rates may be a result of periodic local expansions, and consequent increases in sequence diversity. This property exists in east and west lineages, and in all cases appears to precede diversification of a stable clade.

Previously described YFV sequence data supports ecological features of geographic niche association. 3′ UTRs of YFV contain specific repeat regions that occur with frequencies reflecting the major region of origin [39]. The presence of three noncoding tandem repeat sequence elements in West African YFV isolates, and two in East African isolates, allows a parsimonious interpretation that one repeat segment is gained or lost in lineage diversification between East and West Africa. Discrete model estimates inferred in this study substantiate the longitudinal orientation of African lineage diversification (Figure 3). This finding provides a context to the paradigm of viral transition between contiguous vegetative ecosystems, a geographic proxy measure for human YFV infection risk [18]. The discrete model permits time-reversible transitions, or free lineage exchange between location states without preconditioned directionality. This aspect of the model mirrors the reality of sylvatic YFV circulation, in which epizootic foci are hypothesized to be geographically dynamic [40]. Spatial boundaries represented by sequence features will only be fully resolved by rigorous surveillance.

The observation of positive selection for the envelope protein mutation G100S in the fusion loop has been demonstrated for prM/E alignments of YFV sequence data containing combined taxa of African and South American origin [14], [41]. The significance of the mutation G100S for African YFV emergence dynamics is unknown, but it arises independently three times in our expanded African dataset. Times between isolations of this mutation are seven years (Democratic Republic of Congo/1958 and Guinea Bissau/1965) and 27 years (Guinea Bissau/1965 and Senegal/1992), respectively. Selection at this site would be expected to be biologically significant. In aggregate, experimental evidence suggests that flavivirus envelope protein mutations in the fusion loop play a significant role in antigenicity and host entry. Mutational analysis of this region provided evidence for sequence conservation to facilitate effective fusion with host intracellular membranes [42]. A comparison of flavivirus fusion loop sequences found this mutation to occur mostly in YFV isolates, with rare occurrence in other members of the genus [43]. The apparent mutational diversity at E100 has been found in temporally and geographically diverse YFV isolate groups, but the result of a selection pressure that has not been identified.

Significantly, the results of our BSSVS analysis strongly supports transitions that span the most ancestral node of the inferred tree, providing evidence for connection of the ecosystems of eastern and western circulation. Long-distance (Senegal-Ethiopia, The Gambia-Kenya) transitions were reconstructed from the West to each of the subordinate Eastern clades, implying a level of independence for the east and east/central genotypes for the seeding of virus from unobserved reservoirs. This property contrasts with the transitions reconstructed from the Western lineage, in which local diffusions are supported. Transitions to Angola were not supported by BSSVS significance criteria used in the study. This is not unexpected, considering that transition to Angola from eastern circulation is not consistent with the pattern of east-west diffusions so far described. However, the highest root state probability was estimated to have originated from Angola, implying that the 1971 urban outbreak in Luanda was seeded from a more ancestral population than is represented in the phylogeny to date. The virus Angola/1971 may have entered the population through a rare dispersal mechanism, such as traveler movement or population displacement. Disruptions to population or infrastructure provoked by the Nigerian Civil War (1967–1970) or the Angolan War of Independence (1961–1975) may represent appropriate historical correlations to the outbreak; however this interpretation must considered with caution as is it is based on a single isolation event.

Coalescent demographic analysis indicates stability of the viral population across the most adequately sampled timescale of the dataset (Figure 2). Biologically, this finding supports a classic model of persistent sylvatic arboviral maintenance. Campaigns of reactive mass vaccination presume risk to community members following the appearance of human cases, and is a response to presumed static dynamics of YFV spillover events during response timescales [37], [44]. Irregular East African YFV emergence is a result of human intrusion into the range of viral circulation, including human movement as a consequence of civil unrest (a recent example is a 2005 Sudanese YFV outbreak, of which commentary suggested that previously blocked north/south pastoralist migration routes had opened due to relief in civil tensions, exposing immunologically naive individuals to fatal YFV infections [45]) and with agricultural and population changes brought by the European colonial era [4]. Low sampling frequency preceding 1948 does not permit resolution of demographic trends for the early time interval.

This alternative model of YFV emergence is supported by the estimated posterior density intervals for the divergence dates of east and west lineages, which was estimated to have occurred between 1733 and 1802 CE, suggesting that alterations or intrusions to continental ecology may have been concurrent with European colonization of YFV-endemic regions (Table 1). A plausible reconstruction for this time period of geographic movements is supported by studies on human immunodeficiency virus 2 (HIV-2) that suggest population movements during this time period [46]. Again, flat demographic estimates offer a constant paradigm from which to model variable intrusions of humans into regions of YFV circulation.

Bayes factor support for Cauchy-distributed viral diffusion rates suggests the existence of a heterogenous dispersal process. It is possible that the irregularities seen in emergence of human cases in Central and East Africa are the combined result of wandering epizootic transmission with variability of interaction between susceptible humans and regions of sylvatic maintenance. Forty-three of the 49 isolates used in the study were obtained from human cases, so this property is relatively uniform in the dataset. Local-scale geographic averaging produced by discrete state modeling offers some clarity to this question. By including all isolates within a politically defined landmass, the discrete model may provide some correction to sampling errors introduced by the transit of human cases from the location of infection before discovery of illness.

Estimated connection of the east and west lineages ceased during the 30-year interval starting in 1644 CE (Figure 4). Our modeled reconstruction of YFV lineage phylogeography independently supports topotype mapping derived from ecological data, and expands upon this information to suggest the presence of overlapping foci of circulation [34]. Assuming a centered spatial average for node location estimates, diffusion analysis supports the overlap of East African genotypes in recent history. Posterior diffusion uncertainty was visualized against a publicly available model for predicting the presence of *Ae. africanus*, the major vector of sylvatic YFV in Africa (Figure 4) [33]. Considering the timescales of the diffusion analyses, it is possible that past spatial mixing of the virus obscures aspects of the dispersal path from basal nodes. This is especially true for the root of the inferred phylogeny, which at the timescales represented occupies a central section of the African continent (80%HPD).

The inference of divergence events presented in this study remedies gaps in the historical understanding of YFV emergence, and offers further opportunity to correlate viral dispersal events with human social and demographic history. Evidence for stable maintenance, in a context of irregular emergence, highlights the need for further surveillance and acquisition of YFV sequence information. The results of this study have potential to be incorporated into design of regional surveillance efforts and implementation of vaccination strategies.

## Supporting Information

Figure S1 Neighbor-joining tree phylogeny computed from all publicly available partial sequence data for 3′ untranslated regions of the yellow fever virus genome. Tree was computed using observed distance with 1000 bootstrap replicates, the results of which are displayed as percentage for selected nodes. Genotypes are listed at right.
(PDF)

Figure S2 MCC phylogeny showing relative branch mutation rates annotated to branch colors, under a lognormally distributed clock. Rates are shown on a gradient from blue (lowest) to yellow (highest).
(PDF)

Table S1 **Panel a:** Complete taxon list, including major attributes. Coordinates are listed in decimal degrees and sampling date is listed as inputted to software, in decimal years. Newly presented taxa are highlighted in grey. **Panel b:** Taxa used to infer the neighbor-joining phylogeny of 3′ NS5/untranslated region sequences, presented in Figure S2.
(XLSX)

Table S2 Posterior mean estimates for diffusion model parameters used in the study. 95% HPD intervals are listed in parentheses, if applicable. Bayes factors for marginal likelihood are listed relative to the fixed rate continuous diffusion model. Marginal likelihood estimation was performed by path sampling and stepping stone methods, the results of which were confirmed by two independent runs of 30 million steps for each diffusion model. Results for the first chain are shown.
(PDF)
