# Supplementary material for: Phylogeographic Reconstruction of African Yellow Fever Virus Isolates Indicates Recent Simultaneous Dispersal into East and West Africa
Source: PLoS Negl Trop Dis. 2013 Mar 14;7(3):e1910. doi: 10.1371/journal.pntd.0001910 (PMC3597480; doi:10.1371/journal.pntd.0001910)

Figure S1: Neighbor-joining tree phylogeny computed from all publicly available partial sequence data for 3' untranslated regions of the yellow fever virus genome. Tree was computed using observed distance with 1000 bootstrap replicates, the results of which are displayed as percentage for selected nodes. Genotypes are listed at right.

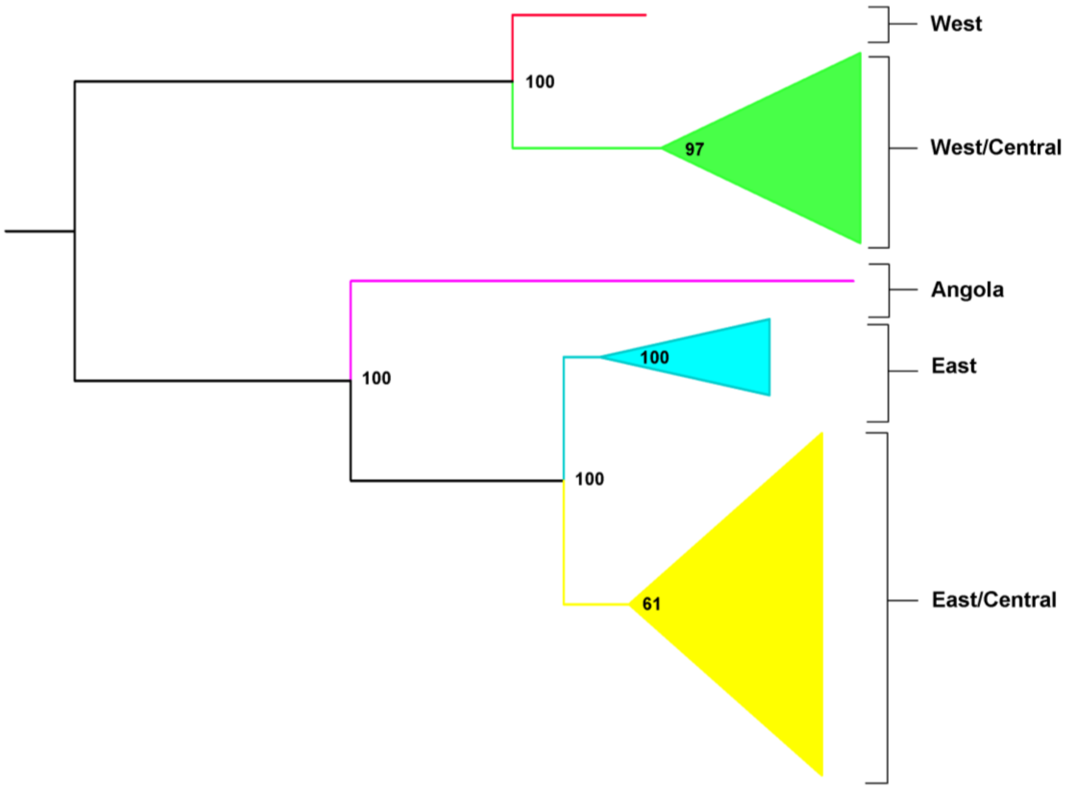

Supplement: Figure S1 — Neighbor-joining tree phylogeny computed from all publicly available partial sequence data for 3′ untranslated regions of the yellow fever virus genome. Tree was computed using observed distance with 1000 bootstrap replicates, the results of which are displayed as percentage for selected nodes. Genotypes are listed at right. (PDF) [file pntd.0001910.s001.pdf]
