# Supplementary material for: Phylogeographic Reconstruction of African Yellow Fever Virus Isolates Indicates Recent Simultaneous Dispersal into East and West Africa
Source: PLoS Negl Trop Dis. 2013 Mar 14;7(3):e1910. doi: 10.1371/journal.pntd.0001910 (PMC3597480; doi:10.1371/journal.pntd.0001910)

Figure S2: MCC phylogeny showing relative branch mutation rates annotated to branch colors, under a lognormally distributed clock, shown in calendar years CE. Rates are shown on a gradient from blue (lowest) to yellow (highest).

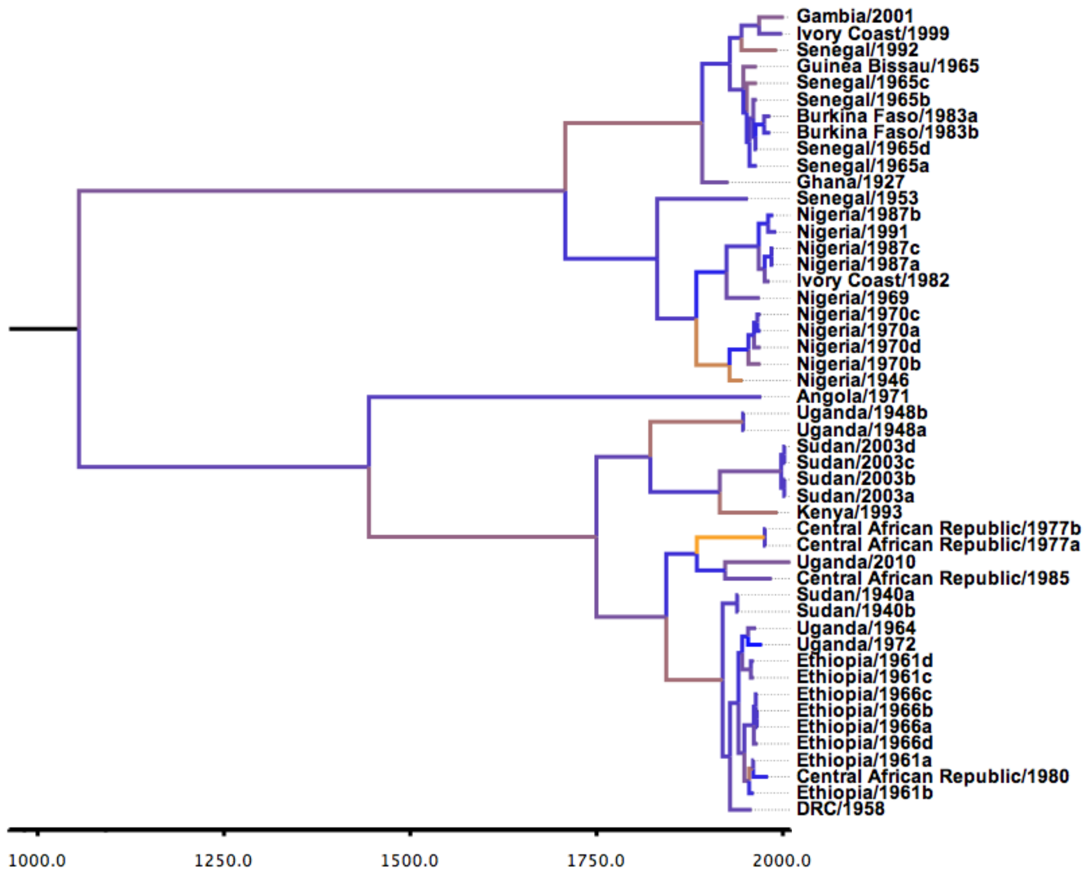

Supplement: Figure S2 — MCC phylogeny showing relative branch mutation rates annotated to branch colors, under a lognormally distributed clock. Rates are shown on a gradient from blue (lowest) to yellow (highest). (PDF) [file pntd.0001910.s002.pdf]
