# Supplementary material for: Phylogeographic Reconstruction of African Yellow Fever Virus Isolates Indicates Recent Simultaneous Dispersal into East and West Africa
Source: PLoS Negl Trop Dis. 2013 Mar 14;7(3):e1910. doi: 10.1371/journal.pntd.0001910 (PMC3597480; doi:10.1371/journal.pntd.0001910)
Supplement: Table S2 — Posterior mean estimates for diffusion model parameters used in the study. 95% HPD intervals are listed in parentheses, if applicable. Bayes factors for marginal likelihood are listed relative to the fixed rate continuous diffusion model. Marginal likelihood estimation was performed by path sampling and stepping stone methods, the results of which were confirmed by two independent runs of 30 million steps for each diffusion model. Results for the first chain are shown. (PDF) [file pntd.0001910.s004.pdf]

Table S2: Posterior mean estimates for diffusion model parameters used in the study. 95% HPD intervals are listed in parentheses, if applicable. Bayes factors for marginal likelihood are listed relative to the fixed rate continuous diffusion model. Marginal likelihood estimation was performed by path sampling and stepping stone methods, the results of which were confirmed by two independent runs of 30 million steps for each diffusion model. Results for the first chain are shown.

**Model**	**ln P(model | data) -Path Sampling**	**Bayes Factor -Path Sampling**	**ln P(model | data) -Stepping Stone**	**Bayes Factor -Stepping Stone**	**TMRCA Mean Age**	**TMRCA Mean Latitude**	**TMRCA Mean Longitude**	**Mean Diffusion Rate (km/yr)**
Continuous	-3952.01	0	-3945.77	0	466.63 (192.31, 802.88)	3.96 (-6.60, 15.12)	9.881 (-15.94, 35.07)	19.19 (10.16, 28.22)
Cauchy	-3734.66	217.35	-3729.34	216.42	733.11 (287.47, 1278.62)	4.32 (-9.03 14.28)	10.50 (-12.53, 34.54)	10.57 (4.86, 17.38)
Lognormal	-3824.14	127.86	-3816.37	129.40	1114.44 (334.91, 2030.73)	4.01 (-10.04, 15.61)	8.85 (-17.33, 34.30)	6.56 (2.33, 11.66)
Gamma	-3823.13	128.88	-3811.03	134.73	879.70 (322.97, 1492.30)	3.89 (-9.08, 15.79)	10.63 (-14.27, 33.59)	8.152 (3.82, 13.73)
